# One Enzyme, Many Faces: The Expanding Role of DPP3 in Cardiovascular and Critical Care

**DOI:** 10.3390/jcm14217459

**Published:** 2025-10-22

**Authors:** Georgios E. Zakynthinos, Nikolaos K. Kokkinos, Ioanna G. Tzima, Ilias E. Dimeas, Ioannis Gialamas, Andreas Gerostathis, Ourania Katsarou, Aikaterini Tsatsaragkou, Konstantinos Kalogeras, Evangelos Oikonomou, Gerasimos Siasos

**Affiliations:** 1 3rd Department of Cardiology, “Sotiria” Chest Diseases Hospital, Medical School, National and Kapodistrian University of Athens, 11527 Athens, Greecekalogerask@yahoo.gr (K.K.);; 2 School of Medicine, University College Dublin, D04 C1P1 Dublin, Ireland; 3 Department of Respiratory Medicine, St. Vincent’s University Hospital, D04 T6F4 Dublin, Ireland; 4 Cardiovascular Division, Brigham and Women’s Hospital, Harvard Medical School, Boston, MA 02115, USA

**Keywords:** dipeptidyl peptidase 3, dipeptidyl peptidase III, DPP 3, DPP III, cardiogenic shock, cardiovascular disease, heart failure, septic shock, critical care, biomarker

## Abstract

Dipeptidyl peptidase 3 (DPP3) is a zinc-dependent aminopeptidase that is found in several places and is thought to be a cytosolic enzyme that helps break down peptides. Recent studies, however, have revealed its extensive therapeutic relevance upon release into circulation, functioning not only as a biomarker for cellular injury but also as an active modulator of cardiovascular homeostasis and critical disease. High levels of circulating DPP3 (cDPP3) have been linked to the causes of cardiogenic shock, septic shock, acute coronary syndromes, heart failure, and serious viral diseases like COVID-19. Its enzymatic breakdown of angiotensin II disrupts vascular tone and myocardial contractility, leading to hemodynamic instability and multi-organ failure. In numerous cohorts, cDPP3 levels reliably correspond with disease severity, acute renal damage, and death, but dynamic trajectories yield superior predictive information relative to single assessments. In addition to risk stratification, translational studies utilizing rodent and porcine models illustrate that antibody-mediated inhibition of cDPP3 with the humanized monoclonal antibody Procizumab reinstates cardiac function, stabilizes renal perfusion, diminishes oxidative stress and inflammation, and enhances survival. First-in-human experiences in patients with refractory septic cardiomyopathy have further emphasized its therapeutic promise. DPP3 is a good example of a biomarker and a mediator in cardiovascular and critical care. Its growing clinical and translational profile makes cDPP3 a strong predictor of bad outcomes and a prospective target for treatment. Ongoing clinical trials using Procizumab will determine if neutralizing cDPP3 can lead to enhanced outcomes in individuals with cardiogenic and septic shock. This review outlines the physiological mechanisms, clinical implications, and emerging therapeutic potential of DPP3 in cardiovascular and critical care. Ongoing trials with Procizumab will clarify whether neutralizing cDPP3 can improve outcomes in patients with cardiogenic and septic shock.

## 1. Introduction

With 17.9 million deaths from cardiovascular disease in 2019, it continues to be the leading cause of death and disability worldwide [1]. Low- and middle-income nations are disproportionately affected, as their rates of early mortality are particularly high [1,2,3,4]. The socioeconomic burden is still increasing, and results are still inadequate despite advancements in prevention and therapy, highlighting the urgent need for innovative therapeutic and diagnostic approaches [3,5,6]. Despite advanced categorization methods and improved clinical therapy, cardiogenic shock (CS), one of the deadliest cardiovascular emergencies, continues to have an in-hospital death rate of 30–50%, with nearly half of survivors requiring readmission and 15% dying within a year.

Similarly to this, despite decades of research, sepsis and septic shock still pose significant global issues due to their high morbidity and mortality rates, underscoring the need for novel biomarkers that might support diagnosis, risk assessment, and customized treatment [7,8].

Dipeptidyl peptidases (DPPs) have garnered more interest in this context. This family of enzymes, which includes eight human members (DPP1–DPP10; DPP5 and DPP11 are bacterial) and controls a variety of biological activities, from cell-cycle regulation and oxidative stress defense to peptide degradation and protein maturation [9]. Dipeptidyl peptidase 3 (DPP3) is an 80–85 kDa zinc-dependent aminopeptidase that normally lives in the cytosol, while recent research indicates that it may also be found in the membrane [10,11,12,13]. It contributes to the metabolism of a number of bioactive peptides by cleaving dipeptides from the N-terminus of oligopeptides [10].

Since Brand and Lefer characterized a low-molecular-weight chemical released during hemorrhagic shock that impairs cardiac function in the 1960s, the idea that a circulating myocardial depressant factor contributes to shock syndromes has existed [14]. DPP3 was discovered decades later as a candidate with remarkably comparable properties: it is produced upon cell death, is broadly distributed in tissues, and may interact with inflammatory pathways [14]. In fact, circulating DPP3 (cDPP3) has become a biomarker of cellular damage, and it is especially important in septic shock and cardiovascular conditions [9].

By degrading angiotensin II, increased cDPP3 mechanistically interferes with vasoconstrictive signaling and blood pressure regulation [10]. Additionally, it weakens compensatory myocardial contractility by having adverse inotropic effects [11]. High cDPP3 levels are consistently associated with negative outcomes in sepsis, septic shock, and cardiogenic shock in clinical trials [7,10,12]. Inhibition of the enzyme is being investigated as a novel therapeutic option because, in addition to its function as a biomarker, there is growing evidence that cDPP3 may actively drive disease development, sustaining circulatory and renal failure [15].

All these results suggest that DPP3 is more than just a passive marker of cellular injury. Instead, it seems to play a significant role as a mediator between critical disease and cardiovascular collapse, which makes it a desirable target for therapeutic intervention as well as risk classification.

The present review aims to synthesize current knowledge on the physiological and pathophysiological roles of DPP3, highlighting its relevance across the cardiovascular continuum and in critical illness. We discuss the molecular mechanisms linking DPP3 to cardiovascular and renal regulation, summarize experimental and clinical evidence of its diagnostic and prognostic significance in heart failure, acute coronary syndromes, cardiogenic and septic shock, and explore emerging data on antibody-mediated inhibition as a potential therapeutic approach. By integrating mechanistic, translational, and clinical perspectives, this review seeks to clarify the multifaceted role of DPP3 and its potential incorporation into future diagnostic and treatment strategies in cardiovascular and critical care medicine.

Despite advanced categorization methods and improved clinical therapy, cardiogenic shock (CS), one of the most deadly cardiovascular emergencies, continues to have an in-hospital death rate of 30–50%, with nearly half of survivors requiring readmission and 15% dying within a year [16,17,18,19,20,21].

## 2. Physiology and Pathophysiology of DPP3: Linking Intracellular Function to Critical Illness

Dipeptidyl peptidase 3 is commonly recognized as a soluble cytosolic enzyme in human cells [11,12]. While predominantly intracellular, research has indicated membrane-associated activity in particular tissues, including the calf brain and other rat organs [13,22]. Under conditions of oxidative stress, DPP3 has been reported to translocate from the cytosol to the nucleus, where it may participate in the cellular stress response and DNA damage protection mechanisms [23,24]. Furthermore, extracellular DPP3 has been detected in human cerebrospinal fluid, sheep CSF, seminal plasma, and retroplacental serum [13,25].

DPP3 belongs to the M49 family of zinc-dependent metallopeptidases, encoded by the DPP3 gene on chromosome 11q12–q13.1 [13]. The enzyme contains the conserved HELLGH zinc-binding motif within its catalytic domain, which coordinates Zn^2+^ for dipeptidyl-peptidase activity, preferentially cleaving Xaa–Pro bonds. Its three-dimensional structure reveals flexible domains that close around substrates, a feature linked to broad substrate specificity [13]. Although DPP3 is expressed ubiquitously, its regulation is not fully characterized. Experimental data suggest that oxidative stress and certain oncogenic signals upregulate DPP3 transcription, aligning with its role in cytoprotection and cellular stress responses. However, details on transcriptional control, post-translational modifications, and degradation remain limited and warrant further study [13].

A primary focus is the correlation between DPP3 and the renin–angiotensin–aldosterone system (RAAS). The RAAS regulates cardiovascular and renal homeostasis via a cascade initiated by the renin-mediated cleavage of angiotensinogen into angiotensin I (ANG I). Angiotensin-converting enzyme (ACE) subsequently produces angiotensin II (ANG II), the principal effector peptide, which induces vasoconstriction, regulates salt and water homeostasis, stimulates sympathetic activity, and enhances positive inotropy and chronotropy [26,27]. These processes are vital for maintaining blood pressure and tissue perfusion; nevertheless, prolonged exposure to ANG II might induce harmful remodeling. Other peptides generated from angiotensin have different effects. For example, ANG III mostly acts like ANG II [27], while ANG (1–7) and ANG IV start other pathways that work against the conventional RAAS axis [28].

DPP3, which is present in very low amounts in healthy people [29,30], can cut up almost all of these angiotensin peptides [13]. When DPP3 is missing in vivo, ANG II, III, IV, and ANG (1–5) levels rise, which causes people to drink more water and puts more stress on their kidneys [31]. In contrast, during stress situations, the increased release of DPP3 hastens the degradation of ANG II, which weakens compensatory vasoconstriction and leads to vasodilatory collapse [13,30,32]. This approach provides a credible rationale for the RAAS anomalies identified in catecholamine-resistant shock, previously attributed mainly to compromised ACE function or AT1R signaling [33].

The mechanism by which intracellular DPP3 enters the circulation remains unclear. Evidence indicates that cell death is crucial: activation of the anti-Fas receptor enhances extracellular DPP3 activity by disrupting the plasma membrane during secondary necrosis [34]. Both apoptotic and necrotic routes in Fas-mediated death have been involved in this manner [35,36]. Clinical evidence corroborates this notion, as escalating cellular damage during shock correlates with significant increases in circulating DPP3 [37].

Quantification of circulating DPP3 (cDPP3) in plasma or serum has been standardized using the luminometric immunoassay (DPP3-LIA) developed by 4TEEN4 Pharmaceuticals (Hennigsdorf, Germany), which has been employed in all major clinical studies to date [29]. This sandwich-type assay uses monoclonal antibodies specific for human DPP3 and enables reliable quantification with high specificity and reproducibility. The method has demonstrated good sample stability after multiple freeze–thaw cycles and prolonged storage at −80 °C [29]. Despite its analytical robustness, it remains uncertain whether total protein concentration or enzymatic activity provides the most clinically relevant information, emphasizing the need for assay harmonization and further validation in critical care settings [29].

In addition to its role in the RAAS, DPP3 contributes to the regulation of oxidative stress through interaction with the Keap1–Nrf2/ARE pathway, a central cellular defense mechanism against oxidative damage [38,39,40]. Under physiological conditions, the transcription factor Nrf2 is sequestered in the cytoplasm by Keap1 (Kelch-like ECH-associated protein 1), which facilitates its ubiquitination and proteasomal degradation. During oxidative stress, this interaction is disrupted, allowing Nrf2 to accumulate and translocate into the nucleus, where it binds to antioxidant response elements (ARE) and induces the expression of antioxidant and cytoprotective genes such as HO-1 and NQO1. DPP3, via its ETGE motif, competes with Nrf2 for Keap1 binding, thereby preventing Nrf2 degradation and promoting its nuclear translocation [41,42,43,44,45,46]. This positions DPP3 as an activator of the cellular antioxidant defense system [47]. Supporting evidence includes observations in dpp3-knockout mice (Appendix A), which exhibit increased ROS levels, impaired Nrf2/HO-1 signaling, and bone loss phenotypes [38]. Furthermore, DPP3 has demonstrated neuroprotective effects: in hippocampal neurons subjected to oxygen–glucose deprivation/reoxygenation, DPP3 overexpression reduced ROS production, decreased apoptosis, and enhanced cell survival [39].

The enzyme also helps control the immune system. DPP3 expression and activity have been shown in both innate immune cells (such polymorphonuclear granulocytes and monocytes) and adaptive lymphocytes [48,49]. Its absence disturbs cytokine equilibrium, resulting in the increase in pro-inflammatory mediators such as TNFα, IL-1β, and IL-6 in deletion animals [39,50]. These results indicate that DPP3 plays a role in regulating both pro-inflammatory and anti-inflammatory pathways, consequently affecting immunological homeostasis.

It is important to note that studies of catalytic efficiency show that DPP3 breaks down enkephalins and endomorphins considerably faster than angiotensins, which suggests that it plays a function in pain processing. Clinical observations associate diminished DPP3 activity in cerebrospinal fluid with acute pain states; nevertheless, this subject is beyond the purview of the current review [13]. Likewise, the enzyme’s possible role in cancer biology has been investigated for decades, with mounting evidence suggesting that DPP3 overexpression may promote carcinogenesis [47,51,52,53,54]. This area is interesting, but it goes beyond the main topics of this paper, which are cardiovascular and critical care.

DPP3 in circulation is particularly pertinent to acute care medicine. Under normal circumstances, levels that are low rise quickly during shock syndromes [29]. In these contexts, ANG II typically maintains perfusion; however, heightened DPP3 activity swiftly destroys ANG II, attenuating this crucial response and potentially reducing the effectiveness of therapeutic ANG II infusion [13]. Experimental models validate the harmful potential of elevated circulating DPP3: intravenous injection diminishes cardiac and renal function, whereas neutralization with a monoclonal antibody reinstates hemodynamic stability, enhances organ performance, and reduces mortality [55,56].

Kinetic evidence indicates that cDPP3 levels rise rapidly following cellular injury, particularly in septic and cardiogenic shock. Both experimental and clinical data demonstrate that DPP3 concentration and enzymatic activity increase proportionally with the severity of shock and are highest in non-survivors [13]. In sepsis, elevated plasma DPP3 promotes excessive degradation of angiotensin II into angiotensin IV, while leaving angiotensin I unaffected—thereby increasing the angiotensin I/II ratio and reducing AT1 receptor stimulation, a mechanism that contributes to circulatory collapse. Inhibition of DPP3 with the monoclonal antibody Procizumab quickly restores left ventricular contractility and improves survival in animal models, supporting a direct hemodynamic role [57]. These findings highlight DPP3 as a fast-acting myocardial depressant factor, whose plasma kinetics mirror the extent of tissue injury and whose effects are dynamically reversible [13]. However, the precise plasma half-life, clearance mechanisms, and in vivo turnover remain incompletely characterized and represent an important target for future kinetic studies.

These data collectively underscore DPP3 as a versatile enzyme involved in peptide metabolism, redox homeostasis, immunological modulation, and circulatory regulation. When it is released into the extracellular space, it changes from a housekeeping protease to a powerful mediator of shock pathophysiology. This makes it both a biomarker and a possible target for treatment.

Beyond its mechanistic and pathophysiological implications, DPP3 has also emerged as a promising biomarker of hemodynamic compromise. Elevated circulating DPP3 concentrations correlate closely with outcomes in cardiogenic, septic, and vasodilatory shock, performing comparably to established prognostic markers such as BNP, cardiac troponins, and lactate. Unlike these conventional markers, which reflect secondary consequences of myocardial strain, necrosis, or hypoperfusion, DPP3 directly participates in disease pathogenesis by degrading angiotensin II and impairing vasomotor tone and cardiac contractility. This dual role—as both a mediator and indicator of circulatory failure—provides unique clinical relevance. Furthermore, the availability of a targeted neutralizing antibody (Procizumab) introduces a potential therapeutic dimension, distinguishing DPP3 from traditional biomarkers and reinforcing its translational importance in critical care (Table 1).

## 3. The Expanding Clinical Role of DPP3 in Heart Disease and Critical Care

### 3.1. Circulating DPP3 as a Marker and Modulator in Heart Failure

Experimental and clinical evidence suggests that DPP3 plays an important role in the progression and prognosis of heart failure. In preclinical work, exogenous administration of recombinant DPP3 reduced angiotensin II–driven myocardial remodeling. Four weeks of intravenous treatment markedly attenuated cardiac fibrosis and offered organ protection in models of angiotensin II–induced injury [58]. Similarly, in type 2 diabetic db/db mice, eight weeks of recombinant DPP3 infusion alleviated inflammatory infiltration, improved diastolic dysfunction, and reduced myocardial fibrosis, although glycemic control was unaffected [59]. Together, these studies indicate that DPP3 can counteract inflammation and fibrosis, thereby preventing the transition to chronic heart failure, even if the precise molecular mechanisms of this protective effect remain uncertain [3].

The clinical significance of cDPP3 has been examined in large patient cohorts. In a prospective study with a median follow-up of 21 months analyzing 2156 serum samples from individuals with worsening heart failure, cDPP3 was measured using a luminometric immunoassay (DPP3-LIA, 4TEEN4 Pharmaceuticals, Hennigsdorf, Germany). Median concentrations were 11.45 ng/mL, ranging from 2.8 to 84.9 ng/mL [60]. Patients with higher cDPP3 levels had elevated renin [120.7 IU/mL, interquartile range (IQR) 34.74–338.9 vs. 78.3 IU/mL, *p* < 0.001] and aldosterone [116 IU/ML vs. 88 IU/mL, *p* < 0.001] concentrations compared to those in lower quartiles. Independent predictors of high cDPP3 included liver enzymes (alanine aminotransferase, bilirubin), the absence of diabetes, and elevated osteopontin, fibroblast growth factor-23 (FGF-23), and NT-proBNP, all with *p* < 0.001 [60].

Stratification by quartiles revealed clinically meaningful differences. Patients in the top quartile (median DPP3: 17.95 ng/mL) were more often in NYHA class IV (13.4% vs. 11.3%, *p* = 0.001), had a higher frequency of previous valvular surgery (12.1% vs. 6.1%, *p* < 0.001), and more commonly showed valvular etiology of heart failure (11.0% vs. 6.9%, *p* = 0.003). Atrial fibrillation was more prevalent in this group (51.6% vs. 43.3%, *p* = 0.001), whereas diabetes mellitus was less frequent (27.5% vs. 33.0%, *p* = 0.020). Men also had higher DPP3 concentrations than women (mean 13.0 vs. 12.2 ng/mL, *p* = 0.010) [60]. However, it should be noted that the absolute differences in cDPP3 concentrations between many of these subgroups were relatively small, and that these patients overall presented with more signs of congestion, elevated liver enzymes, lower cholesterol, and greater neurohormonal activation compared with the lower three quartiles, while being less often treated with ACE inhibitors or ARBs [60].

Outcomes correlated strongly with cDPP3 levels. Mortality increased from 20.4% in the lowest quartile to 36.0% in the highest. Similarly, the combined endpoint of death or hospitalization for heart failure occurred in 34.7% of patients in the lowest quartile compared to 50.3% in the highest [60]. In univariable analyses, higher cDPP3 was associated with both mortality and the combined outcome (*p* < 0.001 for both). However, these associations lost statistical significance after adjustment for other prognostic markers, especially osteopontin, FGF-23, and the BIOSTAT-CHF risk model [60].

Additional data from a separate cohort of 365 patients with stable heart failure with reduced ejection fraction (HFrEF) provide further insight. Median age was 65 years (IQR 54–73), 77% were men, and 54% had ischemic etiology. Median cDPP3 was 11.36 ng/mL, similar to levels in healthy volunteers [61]. No differences were seen between ischemic and non-ischemic patients or across different RAAS-inhibitor treatments. In this stable HFrEF population, cDPP3 above 15 ng/mL predicted increased mortality, aligning with the cut-off observed in BIOSTAT-CHF [61]. While not useful for early disease detection, cDPP3 may therefore identify end-stage HFrEF patients who are otherwise difficult to classify, extending its utility beyond cardiogenic shock into chronic disease stratification [61].

In summary, both preclinical and clinical evidence position cDPP3 as a marker that reflects inflammation, fibrosis, neurohormonal activation, and disease severity in heart failure. Although its prognostic impact is attenuated when adjusted for established biomarkers, elevated cDPP3 consistently marks advanced disease and poor outcome, and experimental models suggest it may even be a therapeutic target.

### 3.2. DPP3 in Hypertension

The ability of DPP3 to hydrolyze angiotensin peptides suggests a potential role in the regulation of the RAAS [62]. In 2016, Pang and colleagues showed that giving DPP3 to mice with angiotensin II–induced hypertension through their tails caused a big drop in blood pressure. This was the first time that this enzyme was shown to have a possible therapeutic role in hypertension [58]. Nonetheless, in the same year, knockout animals produced an apparently contradictory result: the absence of DPP3 did not influence systemic blood pressure when assessed with the tail-cuff method [58]. This difference could be due in part to limitations in the methods used, such as stress-induced changes that are common in tail-cuff measurements. Despite this, the results indicate that compensatory cardiovascular mechanisms may maintain blood pressure homeostasis in the absence of DPP3 [3].

Subsequent investigations have augmented these findings by illustrating that repeated intravenous administration of DPP3 decreases systolic blood pressure while offering improved cardiovascular and renal protection. In hypertensive mice, treatment diminished ventricular hypertrophy and myocardial fibrosis, improved renal function, and reduced albuminuria. It also stopped inflammatory and prothrombotic mediators, namely monocyte chemoattractant protein-1 and plasminogen activator inhibitor-1 [63]. These benefits stem from DPP3′s ability to degrade angiotensin II and angiotensin IV, which alleviates vasoconstriction and obstructs subsequent hypertrophic, inflammatory, and coagulation pathways. These results indicate that DPP3 may function as an adjunctive strategy to conventional RAAS reduction by ACE inhibitors or angiotensin receptor blockers, particularly targeting ongoing cardiovascular and renal injury [63].

Despite this promise, the exploration of DPP3 as an antihypertensive therapy has largely stalled since 2016. There are a lot of things that could be wrong that are making this go slower. To begin with, we do not fully understand how the enzyme operates. DPP3 is usually found in cells, however it can also act outside of cells. We do not know all about how it is released, controlled, or interacts with peptides. Second, translation is still a huge difficulty. Some of the most significant factors that still need to be figured out include bioavailability, dosing, delivery methods, and long-term safety in individuals. Third, most of the information comes from proof-of-concept mouse studies, which have not been tested in other models or real-life situations. Lastly, research objectives in the RAAS domain have frequently prioritized more clearly delineated targets such as ACE2, angiotensin receptor blockers, and DPP IV inhibitors, potentially diverting focus and resources from the therapeutic exploration of DPP3 [63].

Although preclinical data robustly suggest that DPP3 may mitigate hypertension and its related cardiovascular and renal complications, the lack of subsequent studies underscores the need for a more comprehensive mechanistic understanding and translational research before identifying this enzyme as a potential therapeutic target.

### 3.3. cDPP3 in Acute Coronary Syndromes: A Novel Biomarker of Injury and Prognosis

Evidence is emerging that cDPP3 is closely linked to myocardial injury and prognosis in patients with ACS. In a prospective case–control study including 70 ACS patients (mean age 62.5 ± 11 years, 68.6% male) and 48 controls (mean age 61.1 ± 10 years, 66.7% male), cDPP3 levels were measured at 24, 48, and 72 h after symptom onset [64]. Concentrations were significantly higher in ACS patients compared with controls, mirroring the pattern of troponin I. Importantly, cDPP3 emerged as an independent predictor of left ventricular ejection fraction (LVEF), similar in strength to NT-proBNP and troponin I [64]. To date, this represents the first report directly linking DPP3 to myocardial dysfunction in ACS, raising the possibility of integrating the enzyme into novel risk assessment strategies [64].

These findings were extended in the large-scale SPUM-ACS cohort (ClinicalTrials.gov Identifier: NCT01000701), which enrolled 4787 patients between 2009 and 2017 [65]. At admission, median cDPP3 levels were elevated [19.0 ng/mL], then decreased within 12–24 h [17.3 ng/mL] and declined further by discharge [14.0 ng/mL; *p* < 0.001] [65]. Prognostic analyses revealed striking associations with mortality. Patients in the high cDPP3 group had a 4.3-fold greater 30-day mortality risk (adjusted HR 4.29, *p* < 0.001). When cDPP3 was modeled continuously, each doubling of concentration corresponded to an 87% increase in 30-day mortality (HR 1.87, *p* < 0.001) [65].

Longer-term outcomes were consistent: elevated cDPP3 predicted all-cause mortality at one year (adjusted HR 2.42, *p* < 0.001), with an approximately 61% increase in mortality risk per doubling of concentration (HR 1.61, *p* < 0.001) [65]. These associations remained robust after adjustment for the GRACE 2.0 risk score [65]. Furthermore, adding cDPP3 to GRACE 2.0 modestly improved mortality prediction, with significant improvements in discrimination and reclassification metrics (30-day mortality: ΔHarrel’s C + 0.016, *p* = 0.030; one-year mortality: ΔHarrel’s C + 0.010, *p* = 0.046) [65].

Persistence of elevated cDPP3 identified a particularly high-risk subgroup. Patients with sustained high levels had a >10-fold increased 30-day mortality (adjusted HR 13.42, *p* < 0.001) and nearly a six-fold higher 1-year mortality (adjusted HR 5.79, *p* < 0.001) compared with those with lower or declining levels [65]. Moreover, increasing cDPP3 over the course of hospitalization was associated with significantly larger infarct size at 30-day follow-up by cardiac MRI [increase: 56.5 g scar mass vs. decrease: 25.5 g, *p* = 0.016] [65].

Together, these studies consistently demonstrate that cDPP3 rises in the acute phase of ACS, reflects myocardial damage, and independently predicts both short- and long-term mortality. Persistence of high cDPP3 identifies patients at especially high risk of death and adverse remodeling, supporting its potential role as a novel biomarker for early risk stratification in ACS.

### 3.4. cDPP3 in Cardiogenic Shock: From Experimental Insights to Clinical Prognosis

Cardiogenic shock represents the clinical scenario in which cDPP3 has been most extensively investigated, and findings from both animal experiments and patient cohorts support its role as a mediator of organ dysfunction and a prognostic biomarker.

In murine models, intravenous administration of recombinant DPP3 induced acute myocardial depression, seen by a reduction in shortening fraction of −10 ± 2%, and compromised renal hemodynamics, indicated by a +0.30 ± 0.02 increase in renal resistive index [56]. These studies demonstrate that elevated extracellular DPP3 can directly impair cardiac contractility and renal function. The observation that DPP3 levels dynamically change during acute ischemic syndromes has fueled interest in its potential role in the pathogenesis of contractile failure and cardiogenic shock [14].

In a prospective cohort of 174 patients with CS, 90-day mortality reached 41%. Admission cDPP3 levels were significantly higher in non-survivors compared with survivors [42.9 ng/mL vs. 26.5 ng/mL, *p* = 0.0002], and elevated concentrations at admission predicted poor outcome [54]. Importantly, rapid declines in cDPP3 during the first 24 h were linked to improved renal function, less need for cardiovascular support, and better survival (*p* < 0.0001), whereas patients with initially low but subsequently rising values had sharply increased mortality [56].

These results align with observations from the SPUM-ACS cohort. In this study, patients who acquired in-hospital CS exhibited significantly elevated cDPP3 levels upon presentation. Every doubling of plasma concentration correlated with a 49% heightened risk of CS (adjusted HR 1.49, *p* = 0.004), and individuals exhibiting elevated concentrations faced over double the risk relative to those with lower levels (adjusted HR 2.15, *p* = 0.008) [65].

Ancillary analyses of the OptimaCC trial, which included 57 patients with CS following acute myocardial infarction, further highlighted the discriminative power of cDPP3. Median baseline concentrations were substantially higher in patients with refractory CS (76.1 ng/mL) than in non-refractory cases (32.8 ng/mL, *p* = 0.014). Admission cDPP3 predicted refractory shock with an AUC of 0.73, outperforming the SOFA score (AUC 0.61) and comparable to lactate (AUC 0.71) [66]. Patients with initially high but rapidly decreasing cDPP3 within 24 h had substantially lower risks of refractory shock and death, regardless of whether norepinephrine or epinephrine was used as vasopressor therapy [66].

Additional prospective work from Italy, including 15 patients with CS admitted to intensive care, confirmed that high cDPP3 correlates with worse hemodynamics and survival. Patients requiring mechanical ventilation had significantly higher levels [40.6 ± 30.9 ng/mL vs. 23.8 ± 4.6 ng/mL, *p* < 0.001], as did those with pulmonary hypertension [64]. Admission values were almost three-fold higher in non-survivors (63.8 ± 54.2 ng/mL vs. 25.4 ± 6.3 ng/mL), and concentrations remained elevated throughout the intensive care unit (ICU) stay (*p* < 0.001) [67].

The ACCOST-HH study (2025) yielded the latest insights by integrating cDPP3 data into an interventional framework. In a cohort of 150 patients with CS, the median baseline values were 43.2 ng/mL, with 52% surpassing the established threshold of 40 ng/mL. High baseline levels correlated with elevated 30-day mortality (adjusted HR 1.7) and a reduced number of days alive without cardiovascular assistance (median 3 vs. 21 days, *p* < 0.0001), and greater need for renal replacement therapy (56% vs. 22%, *p* < 0.0001) and mechanical ventilation (90% vs. 74%, *p* = 0.04) [68]. Notably, prognosis was dramatically influenced by biomarker kinetics: patients with persistently high cDPP3 had a 74% 30-day mortality rate, while those with high initial but rapidly decreasing concentrations had survival comparable to patients with sustained low levels (adjusted HR 0.17, *p* < 0.0001) [68].

In both experimental and clinical research, cDPP3 has repeatedly been identified as a marker for acute myocardial depression and a prognostic indicator in cardiac surgery. High and long-lasting levels are linked to poor survival and failure of several organs, while quick clearance is significantly linked to recovery. These findings identify cDPP3 as both a predictive biomarker and a prospective therapeutic target in the treatment of cardiogenic shock. Table 2 shows all Key Studies on DPP3 in Cardiovascular Diseases.

### 3.5. DPP3 in Septic Shock and Critical Illness: A Dynamic Biomarker of Organ Failure and Mortality

The function of cDPP3 in critically ill patients, especially those with sepsis and septic shock, has gained significant recognition in the past decade.in critically ill patients, particularly those with sepsis and septic shock, has been increasingly recognized over the last decade. Across multiple prospective cohorts, elevated cDPP3 has been consistently linked to organ dysfunction, acute kidney injury (AKI), and mortality, with dynamic changes in biomarker levels carrying important prognostic information.

In a prospective study of 650 intensive care patients, cDPP3 concentrations measured on days 1 and 2 after admission were independently associated with 28-day mortality [HR 1.36, *p* = 0.043; HR 1.49, *p* = 0.002, respectively], while day 3 values were not predictive [69]. Associations with AKI were strong and consistent across all three days, with odds ratios ranging from 1.31 to 1.87. Median cDPP3 levels were highest on admission [56.2 ng/mL], declining on days 2 and 3 (25.7 and 30.1 ng/mL, respectively; *p* < 0.001). Importantly, cDPP3 at day 2 added significant predictive value for mortality to established severity scores such as SOFA and APACHE II [69].

At the point of first medical contact, elevated cDPP3 also carried prognostic weight. In 336 critically ill patients triaged as category 1 emergencies in an Italian ED, non-survivors had significantly higher levels than survivors [43.9 vs. 35.2 ng/mL, *p* < 0.006]. A cut-off of 40 ng/mL doubled the risk of 28-day mortality (HR 2.06), with especially strong predictive power for 24 h mortality (AUC 0.83) [70]. Of note, patients with elevated cDPP3 who were also on ACE inhibitors had the highest mortality in the cohort (37.1%) [70].

Subgroup analysis of the FROG-ICU study assessed 665 patients admitted with shock (422 septic, 136 cardiogenic, 107 hemorrhagic). Overall, 28-day and 1-year mortality were 27% and 47%, respectively. Median baseline cDPP3 was significantly higher in non-survivors [27.0 vs. 18.5 ng/mL, *p* < 0.001]. Mortality risk rose sharply for patients above the highest quintile (>38.9 ng/mL), with HRs of 3.3 for septic shock, 3.3 for cardiogenic shock, and 2.4 for hemorrhagic shock [71]. Higher baseline levels also predicted AKI and the need for renal replacement therapy [71].

AdrenOSS-1 trial recruited 585 ICU patients with severe sepsis or septic shock across 24 centers. Admission cDPP3 was strongly associated with 28-day mortality (adjusted HR 1.5) and with worsening renal and hepatic SOFA subscores [30]. Patients with levels >40 ng/mL who decreased below this threshold after 24 h had improved organ function and outcomes, while persistently elevated concentrations predicted multi-organ failure and high mortality. Patients with rising values from low to high between admission and 24 h (LH group) also had significantly worse outcomes than those who remained low (LL), underscoring the importance of cDPP3 kinetics [30].

The VICTAS trial further explored RAAS disturbances in septic patients with high versus low renin levels. Despite elevated renin, angiotensin II concentrations were not increased. Instead, increased ACE2 and DPP3 expression were observed, with serum DPP3 higher in both normal- and high-renin sepsis groups compared to controls [72]. The ratio of ACE to DPP3 was strikingly inverted between controls and septic patients (<0.1), suggesting that excess DPP3 may contribute to impaired angiotensin II responses in sepsis [72].

The ongoing DARK-Sepsis trial (ClinicalTrials.gov NCT05824767) is designed to examine whether baseline renin and DPP3 levels can predict response to angiotensin II therapy compared with standard vasopressor management in patients with vasodilatory shock requiring norepinephrine [73]. Outcomes include vasopressor requirements, AKI, mechanical ventilation, and mortality, though results are not yet published.

cDPP3 has also been evaluated in patients with severe viral critical illness. In a post hoc analysis of the ACTIV-4 Host Tissue trial, 184 patients hospitalized with COVID-19 and acute hypoxemia were analyzed. Individuals with cDPP3 above the median had significantly higher rates of vasopressor initiation (28.4% vs. 16.7%, *p* = 0.031) and 28-day mortality (25% vs. 6.7%, *p* < 0.001). After adjustment, high cDPP3 remained associated with shorter time to shock, fewer vasopressor-free days, and higher risk of hypotensive events, while renin did not show significant associations [74].

In another multicenter cohort of 80 COVID-19 ICU patients, admission cDPP3 was 35.7 ng/mL. Non-survivors had progressively higher values over days 1, 3, and 7, with AUROCs improving from 0.69 to 0.81. At day 7, combining cDPP3 (>40 ng/mL) with bio-ADM (>70 pg/mL) markedly improved risk prediction for 28-day mortality (HR 11.8, *p* < 0.001) [75].

Finally, in the AKIKI-2 trial ancillary study of patients with severe stage-3 AKI on mechanical ventilation and/or vasopressors, neither cDPP3 nor other biomarkers reliably predicted the short-term need for renal replacement therapy [76].

**Figure 1:**
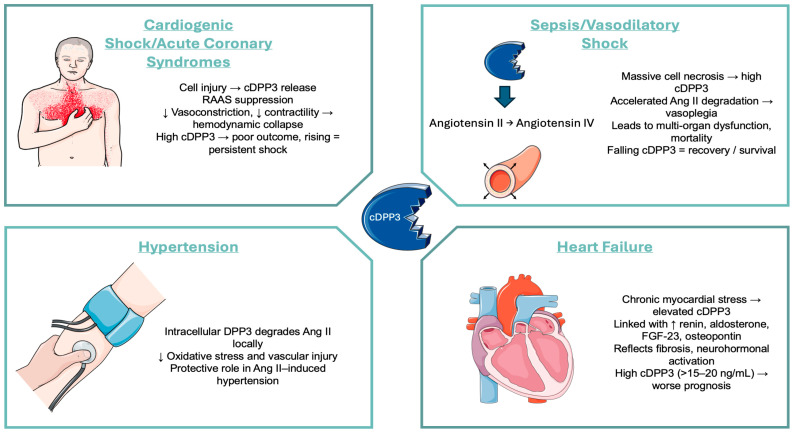
Mechanistic Overview of cDPP3 Activity Across Major Cardiovascular and Critical Illness Scenarios. Image(s) provided by Servier Medical Art (https://smart.servier.com), licensed under CC BY 4.0 (https://creativecommons.org/licenses/by/4.0/).

Across ICU, ED, sepsis, shock, and COVID-19 populations, cDPP3 consistently identifies patients at higher risk of mortality and organ failure, especially when levels are persistently elevated. Kinetic changes—particularly rapid decreases from high to low levels—are strongly associated with recovery, while rising or sustained high levels predict poor outcomes. Together, these findings highlight cDPP3 as a dynamic biomarker of critical illness with prognostic and potentially therapeutic relevance. Table 3 summarizes the studies mentioned above (Figure 1).

## 4. Therapeutic Potential of DPP3 Blockade

Given the accumulating evidence that cDPP3 contributes directly to hemodynamic instability and multi-organ failure, the concept of pharmacological inhibition has attracted major attention. The most advanced candidate, Procizumab (PCZ), is a humanized monoclonal IgG1 antibody specifically designed to neutralize cDPP3 [13].

Preclinical investigations in mice showed that neutralizing cDPP3 with PCZ quickly brought cardiac contractility and renal hemodynamics back to normal while also lowering oxidative stress and inflammatory signaling [56]. These findings confirmed cDPP3 as more than a passive biomarker, underscoring its active involvement in circulatory collapse.

In the cecal ligation and puncture rat model of polymicrobial sepsis, PCZ produced rapid and striking hemodynamic improvements. Left ventricular shortening fraction rose from 39 ± 4% to 51 ± 2% within 30 min (*p* = 0.004), accompanied by higher cardiac output (152 ± 33 vs. 97 ± 25 mL/min, *p* = 0.0079) and stroke volume (0.5 ± 0.1 vs. 0.3 ± 1.0 mL, *p* = 0.009) compared with untreated septic controls [77]. Plasma cDPP3 activity fell sharply in PCZ-treated animals (138 ± 70 vs. 735 ± 255 U/L, *p* = 0.048), while myocardial oxidative stress was significantly reduced (13.3 ± 8.2 vs. 6.2 ± 2.5 units, *p* = 0.005). Short-term survival also improved (83% vs. 63%, *p* = 0.0026), confirming that antibody-mediated inhibition can directly translate into better outcomes in sepsis [77].

In a porcine model of peritonitis-induced septic shock, PCZ treatment reduced circulating cDPP3 and lowered norepinephrine and fluid requirements while maintaining comparable organ perfusion to standard therapy. PCZ animals showed less myocardial injury, higher PaO_2_/FiO_2_ ratios, and lower systemic lactate levels. Mechanistically, antibody treatment was associated with elevated circulating angiotensin II, greater myocardial AT1 receptor expression, and reduced myocardial interleukin-6 mRNA, suggesting restoration of RAAS balance and suppression of inflammatory injury [57].

An accompanying editorial emphasized that high circulating DPP3 consistently associates with adverse outcomes and that exogenous DPP3 produces a rapid, reversible negative inotropic and hypotensive effect in mice [78]. Conversely, antibody-mediated inhibition restores contractility and renal function. Interestingly, these hemodynamic effects depend on RAAS activation: hypotension was observed in angiotensin II-infused hypertensive mice but not in healthy or noradrenaline-driven hypertensive models [78]. The commentary also highlighted apparent discrepancies between pharmacological inhibition and genetic deletion studies regarding oxidative stress. Specifically, while antibody-mediated DPP3 blockade reduces oxidative damage and improves organ function, DPP3-knockout mice exhibit increased ROS accumulation and impaired Nrf2/HO-1 signaling, suggesting that acute enzymatic inhibition in pathological states may have protective effects, whereas lifelong genetic absence disrupts redox homeostasis. This contrast underscores the complexity of DPP3’s physiological roles and the need for further mechanistic clarification [78].

Compassionate use of PCZ in three critically ill patients with refractory septic cardiomyopathy was recently reported from the University Medical Center Hamburg-Eppendorf [79]. All patients presented with extremely high DPP3 activity (≥99th percentile of population values). A single intravenous dosage of PCZ (10 mg/kg over 2 h) was well tolerated, with no adverse effects attributable to the medication. Within 48 h, all three patients showed signs of shock reversal, including lower norepinephrine needs, normal lactate levels, better oxygenation, and improved kidney function. Inflammatory activity also declined, with interleukin-6 falling from a median of 893.5 ng/L to 27.2 ng/L and C-reactive protein decreasing from 298 mg/L to 179 mg/L [79]. These cases provided first-in-human proof of concept for cDPP3 inhibition.

In 2025, the PRO-CARD 1b experiment (NCT06832722) began as a Phase 1/2 research with 130 patients who had cardiogenic shock due to either acute coronary syndromes or bacterial causes. The trial’s goal is to find the best Phase 2 dose of PCZ and to find out how safe, tolerable, pharmacokinetics, and pharmacodynamics it is. Along with traditional shock classifications (vasopressor-dependent hypotension, lactate ≥ 2.0 mmol/L), the inclusion criteria call for high cDPP3. Primary results are expected in 2026 (PRO-CARD 1b protocol).

Together, preclinical and early clinical studies (Table 4) strongly suggest that cDPP3 is not only a biomarker of poor prognosis but also a therapeutic target. Inhibition with Procizumab reverses hemodynamic instability, improves organ function, and reduces inflammatory injury in multiple models. While first-in-human experience is limited, ongoing clinical trials will determine whether cDPP3 blockade can become a novel therapeutic strategy in cardiogenic and septic shock.

## 5. Conclusions

Dipeptidyl peptidase 3 has transitioned from relative obscurity as a cytosolic peptidase to a pivotal entity at the intersection of cardiovascular control and critical illness. Previously considered a passive indicator of cellular injury, cDPP3 is now recognized to have direct pathophysiological effects, such as the degradation of angiotensin II, the impairment of vasomotor tone, the reduction in cardiac contractility, and the facilitation of multi-organ failure.

In a broad range of situations, cDPP3 has shown reliable prognostic significance. In heart failure, elevated amounts are associated with inflammation, fibrosis, and poorer outcomes, especially when levels above 15–20 ng/mL. In hypertension, animal models indicate that DPP3 might mitigate angiotensin II–induced vascular injury, suggesting a dual function as both a biomarker and a possible therapeutic target. In acute coronary syndromes, increased cDPP3 predicts bigger infarct size and is independently associated with both short- and long-term mortality. The most reliable clinical data to date is from cardiogenic shock, where admission cDPP3 levels and their changes over the first 24 to 72 h are very useful for predicting outcomes: high or rising levels mean bad outcomes, while levels that drop quickly mean survival and organ recovery. In sepsis and other critical conditions, cDPP3 independently predicts mortality and AKI, with kinetic assessments being more informative than static ones. These relationships are significant as they encompass viral severe illnesses, including COVID-19, underscoring the extensive applicability of this biomarker.

In addition to risk categorization, translational research has established DPP3 as a therapeutic target. Blocking with the monoclonal antibody Procizumab brings back heart and kidney function, lowers oxidative stress, and increases lifespan in preclinical models of heart failure and sepsis. Compassionate first-in-human usage in refractory shock exhibited safety and favorable hemodynamic responses, facilitating the continuation of Phase 1/2 clinical research.

These data collectively substantiate the concept that DPP3 has “many faces”: a mechanistic factor in cardiovascular collapse, a prognostic biomarker across several critical conditions, and a prospective therapeutic target. The upcoming challenge will be to incorporate cDPP3 measurement into clinical decision-making, validate its application in extensive multicenter trials, and ascertain whether pharmacological inhibition can lead to enhanced outcomes for patients with life-threatening cardiovascular and critical care syndromes.

Despite the substantial progress in understanding DPP3, several knowledge gaps remain. The precise mechanisms governing DPP3 release, clearance, and regulation in vivo are still incompletely defined, as are the determinants of its plasma kinetics under different hemodynamic conditions. Establishing standardized assay thresholds and identifying clinically meaningful cut-off values across diverse patient populations will be crucial for clinical implementation. Furthermore, although Procizumab represents a breakthrough in specific DPP3 inhibition, alternative small-molecule or peptide-based inhibitors have yet to be developed. Future research should focus on large multicenter validation studies, the integration of cDPP3 into prognostic scoring systems, and the exploration of its role in guiding personalized therapy. Such efforts will determine whether targeting DPP3 can move beyond biomarker discovery to become a therapeutic strategy that improves patient outcomes in cardiovascular and critical care medicine.

## Figures and Tables

**Table 1:** Comparison of DPP3 with Established Biomarkers of Hemodynamic Compromise.

Feature	DPP3 (Dipeptidyl Peptidase 3)	BNP/NT-proBNP	Cardiac Troponins (cTnI/cTnT)	Lactate
**Origin/release trigger**	Cytosolic metallopeptidase released after cell injury or necrosis, especially in cardiogenic and septic shock	Secreted by ventricular myocytes in response to increased wall stress	Released from cardiomyocytes after irreversible injury (necrosis or membrane rupture)	Produced by peripheral tissues under anaerobic metabolism and impaired perfusion
**Primary pathophysiological link**	Directly modulates RAAS by degrading Ang II → reduces vasoconstriction and cardiac contractility	Reflects hemodynamic overload and ventricular wall stress	Reflects myocardial ischemia/necrosis	Reflects tissue hypoxia and global perfusion deficit
**Kinetics/dynamics**	Rises rapidly (within hours) in acute shock; declines if tissue injury or hemodynamics improve	Rises within hours of wall stress; declines slowly (½-life ~20 h)	Rises within 2–3 h, peaks at 12–24 h; prolonged if renal failure	Rises within minutes; normalizes rapidly with resuscitation
**Prognostic value**	High levels correlate with mortality, vasopressor need, and organ failure in cardiogenic, septic, and vasodilatory shock	Strong predictor of heart failure severity and outcome	Predictor of infarct size, mortality, and outcomes in ACS or shock	Marker of tissue hypoperfusion and mortality across shock types
**Analytical method**	Luminometric immunoassay (DPP3-LIA, 4TEEN4 Pharmaceuticals)—specific for protein concentration	Electrochemiluminescence or immunofluorescence assays	High-sensitivity immunoassays	Enzymatic or blood-gas analyzers
**Clinical advantages**	Provides mechanistic insight into loss of Ang II-mediated vasoconstriction and myocardial depression; potentially actionable via Procizumab inhibition	Well-validated for chronic HF; limited mechanistic specificity	Gold standard for MI diagnosis; less informative in non-ischemic shock	Rapid bedside use; poor specificity for cause
**Limitations**	Novel biomarker, limited assay availability and reference ranges; uncertain clearance kinetics	Affected by age, renal function, obesity	Elevated in non-ischemic injury, CKD	Easily affected by sampling errors and metabolic conditions

**Table 2:** Key Studies on DPP3 in Cardiovascular Diseases.

Study (Ref)	Population/Setting	Sample Size	Key Findings on cDPP3	Outcomes Associated
**Heart Failure**				
Komeno M et al. [59]	Mice—Heart Failure		Recombinant DPP3 infusion reduced angiotensin II–driven cardiac fibrosis, inflammation, and diastolic dysfunction without affecting glycemia.	Demonstrated protective effects against myocardial remodeling and fibrosis.
Boorsma EM et al. [60]	Human—Heart Failure (BIOSTAT-CHF cohort)	2156	Median cDPP3 11.45 ng/mL. Higher quartiles showed increased renin, aldosterone, liver enzymes, and neurohormonal activation.	Mortality rose from 20.4% (lowest quartile) to 36.0% (highest). Elevated cDPP3 predicted adverse outcomes but lost significance after multivariable adjustment.
Pavo N et al. [61]	Human—Heart Failure	365	Median cDPP3 11.36 ng/mL. Higher levels (>15 ng/mL) predicted worse prognosis.	Elevated cDPP3 associated with mortality in end-stage HFrEF, supporting its role as a marker of advanced disease.
**Hypertension**				
Pang X et al. [58]	Mice—Hypertension		DPP III remarkably reduced blood pressure in Ang II–infused hypertensive mice without alteration of heart rate. DPP III did not affect hemodynamics in noradrenalin-induced hypertensive mice or normotensive mice, suggesting specificity for Ang II.	Demonstrated antihypertensive role and also protective effects against myocardial remodeling and fibrosis.
**Acute Coronary Syndromes**				
Ozden O et al. [64]	Human—Acute Coronary Syndromes	70 ACS, 48 controls	cDPP3 elevated in ACS vs. controls; correlated with troponin I and LVEF.	Identified as an independent predictor of left ventricular dysfunction.
Wenzl FA et al. [65]	Human—Acute Coronary Syndromes (SPUM-ACS)	4787	Admission cDPP3 elevated [median 19.0 ng/mL], declined over hospitalization.	High cDPP3 predicted 30-day and 1-year mortality. Persistent elevation identified highest-risk patients.
**Cardiogenic Shock**				
Deniau B et al. [56]	Human and Mice—Cardiogenic Shock (CardShock study)	174 (Human)	cDPP3 levels were associated with an increased short-term mortality risk. DPP3 induced myocardial depression and impaired kidney hemodynamics in healthy mice.	Decrease in cDPP3 in cardiogenic shock patients within 24h of admission was associated with a favorable outcome
Takagi K et al. [66]	Human—Cardiogenic Shock (OptimaCC trial)	57	Median cDPP3 higher in refractory CS [76.1 vs. 32.8 ng/mL, *p* = 0.014].	Predicted refractory shock; decreasing levels correlated with survival.
Innelli P et al. [67]	Human—Cardiogenic Shock (Italian ICU)	15	Higher cDPP3 in mechanically ventilated and non-survivor patients.	Persistently elevated levels linked to multiorgan failure and death.
Picod A et al. [68]	Human—Cardiogenic Shock (ACCOST-HH trial)	150	Median baseline cDPP3 43.2 ng/mL; >40 ng/mL threshold indicated high risk.	Persistently high cDPP3 → 74% 30-day mortality; rapid decline → improved survival.

Abbreviation list: ACE—Angiotensin-Converting Enzyme, ACS—Acute Coronary Syndrome, Ang II—Angiotensin II, ARB—Angiotensin Receptor Blocker, BIOSTAT-CHF—Biomarker Study to Tailored Treatment in Chronic Heart Failure, cDPP3—Circulating Dipeptidyl Peptidase 3, CS—Cardiogenic Shock, DPP3—Dipeptidyl Peptidase 3, EF—Ejection Fraction, FGF-23—Fibroblast Growth Factor 23, HFrEF—Heart Failure with Reduced Ejection Fraction, HR—Hazard Ratio, ICU—Intensive Care Unit, IQR—Interquartile Range, LVEF—Left Ventricular Ejection Fraction, NT-proBNP—N-terminal pro-B-type Natriuretic Peptide, RAAS—Renin–Angiotensin–Aldosterone System, SPUM-ACS—Special Program University Medicine–Acute Coronary Syndrome.

**Table 3:** Key Studies on cDPP3 in Septic Shock and Critical Illness.

Study (Ref)	Population/Setting	Sample Size	Key Findings on cDPP3	Outcomes Associated
**ICU prospective cohort [69]**	General ICU patients, prospective	650	Admission median 56.2 ng/mL, declined over time. Day 1–2 levels associated with mortality (HR 1.36–1.49) and AKI (OR 1.31–1.87). Day 2 remained independent predictor after adjustment.	28-day mortality; AKI (all stages)
**ED cohort, Rome [70]**	ED triage code 1 (critical) patients	336	Non-survivors had higher levels (43.9 vs. 35.2 ng/mL). cDPP3 > 40 ng/mL doubled 28-day mortality risk (HR 2.06). Strong predictive power for 24 h mortality (AUC 0.83).	24 h and 28-day mortality; interaction with ACE-I use
**FROG-ICU substudy [71]**	Shocked ICU patients (64% septic, 20% cardiogenic, 16% hemorrhagic)	665 (of 2087)	Baseline cDPP3 higher in non-survivors (27.0 vs. 18.5 ng/mL, *p* < 0.001). Highest quintile > 38.9 ng/mL linked to higher mortality in all shock types.	28-day and 1-year mortality; AKI; RRT need
**AdrenOSS-1 [30]**	Severe sepsis/septic shock, multinational ICU	585	Admission cDPP3 associated with mortality (adj. HR 1.5). Persistently high or rising levels → organ failure; falling levels > 40 → improved outcomes.	28-day mortality; organ failure; need for support
**VICTAS substudy [72]**	Sepsis patients (renin stratified)	Subset	High renin sepsis: DPP3 elevated, ACE:DPP3 ratio reversed (<0.1 vs. 29 in controls). Suggests DPP3 contributes to impaired Ang II responses.	Mortality risk linked to renin–DPP3 imbalance
**DARK-Sepsis trial [73]**	Vasodilatory shock, AT2 vs. SOC therapy	Planned 40	Biomarkers: renin and DPP3 measured. Aim to predict vasopressor response. Results pending.	Primary: vasopressor response; secondary: AKI, ventilation, mortality
**ACTIV-4 Host Tissue (COVID-19) [74]**	COVID-19, hypoxemic, no vasopressors at baseline	184	High cDPP3 (>median) → more vasopressor use (28.4% vs. 16.7%, *p* = 0.031) and higher 28-day mortality (25% vs. 6.7%). Renin not predictive.	Vasopressor initiation; 28-day mortality
**COVID-19 ICU cohort [75]**	ICU COVID-19 patients (NL/France)	80	Admission median 35.7 ng/mL. Non-survivors had higher values across days 1–7. Day 7 AUROC 0.81. Combination with bio-ADM (>70 pg/mL) strongly predictive (HR 11.8).	28-day mortality; improved prediction with bio-ADM
**AKIKI-2 ancillary [76]**	Severe AKI (stage 3), ventilated/vasopressor	Subset	cDPP3 did not reliably predict need for RRT within 72 h.	RRT initiation (no added value)

Abbreviation list: ACE-I—Angiotensin-Converting Enzyme Inhibitor, AKI—Acute Kidney Injury, Ang II—Angiotensin II, AUROC—Area Under the Receiver Operating Characteristic Curve, bio-ADM—Bioactive Adrenomedullin, cDPP3—Circulating Dipeptidyl Peptidase 3, CI—Confidence Interval, COVID-19—Coronavirus Disease 2019, ED—Emergency Department, HR—Hazard Ratio, ICU—Intensive Care Unit, NL—Netherlands, OR—Odds Ratio, RRT—Renal Replacement Therapy, SOC—Standard of Care.

**Table 4:** Preclinical and Clinical Studies on Procizumab (PCZ) for cDPP3 Inhibition.

Model/Study (Ref)	Population/Setting	Intervention	Key Findings
**Mouse model [56]**	Acute heart failure mice	PCZ administration	Normalized cardiac contractility, improved renal hemodynamics, reduced oxidative stress and inflammation.
**Rat CLP sepsis model [77]**	36 rats (sepsis via cecal ligation and puncture)	PCZ vs. PBS	Restored LV shortening fraction (39→51%, *p* = 0.004); higher CO and SV; reduced plasma DPP3 activity (138 vs. 735 U/L); improved survival (83% vs. 63%).
**Porcine peritonitis model [57]**	16 anesthetized pigs with septic shock	PCZ vs. standard therapy	Lower norepinephrine and fluid needs; reduced myocardial injury and IL-6 expression; higher PaO_2_/FiO_2_; restored angiotensin II signaling; lower lactate.
**First-in-human case series [79]**	3 critically ill patients with refractory septic cardiomyopathy	Single PCZ dose (10 mg/kg IV)	Safe and well tolerated; shock reversal, reduced norepinephrine, normalized lactate, improved renal/respiratory function, IL-6 fell from 893.5→27.2 ng/L.
**PRO-CARD 1b Trial (NCT06832722)**	Planned 130 patients with CS (ACS or sepsis)	PCZ (Phase 1/2 study)	Evaluating safety, tolerability, PK/PD, and optimal Phase 2 dose. Results expected 2026.

Abbreviation list: ACS—Acute Coronary Syndrome, CLP—Cecal Ligation and Puncture, CO—Cardiac Output, CS—Cardiogenic Shock, DPP3—Dipeptidyl Peptidase 3, IL-6—Interleukin-6, IV—Intravenous, LV—Left Ventricle, LVSF—Left Ventricular Shortening Fraction, PaO_2_/FiO_2_—Arterial Oxygen Partial Pressure to Fraction of Inspired Oxygen Ratio, PBS—Phosphate-Buffered Saline, PCZ—Procizumab, PK/PD—Pharmacokinetics/Pharmacodynamics, SV—Stroke Volume.

## Data Availability

No new data were created or analyzed in this study.

## Abbreviations

The following abbreviations are used in this manuscript:

ACE	Angiotensin-Converting Enzyme
ACE-I	Angiotensin-Converting Enzyme Inhibitor
ACE2	Angiotensin-Converting Enzyme 2
ACS	Acute Coronary Syndrome
AKI	Acute Kidney Injury
APACHE-II	Acute Physiology and Chronic Health Evaluation II
ARE	Antioxidant Response Element
AT1R	Angiotensin II Receptor Type 1
AUC	Area Under the Curve
bio-ADM	Bioactive Adrenomedullin
BNP	B-type Natriuretic Peptide
CI	Confidence Interval
CLP	Cecal Ligation and Puncture
CO	Cardiac Output
COVID-19	Coronavirus Disease 2019
CS	Cardiogenic Shock
cDPP3	Circulating Dipeptidyl Peptidase 3
DB/db mice	Diabetic (leptin receptor-deficient) mouse model
DHE	Dihydroethidium (marker of oxidative stress)
DPP3	Dipeptidyl Peptidase 3
ED	Emergency Department
EF	Ejection Fraction
GRACE	Global Registry of Acute Coronary Events
HF	Heart Failure
HFrEF	Heart Failure with Reduced Ejection Fraction
HR	Hazard Ratio
hs-TnT	High-Sensitivity Troponin T
ICU	Intensive Care Unit
IgG1	Immunoglobulin G1
IL-6	Interleukin-6
IQR	Interquartile Range
IV	Intravenous
LRT	Likelihood Ratio Test
LVEF	Left Ventricular Ejection Fraction
LVSF	Left Ventricular Shortening Fraction
MAP	Mean Arterial Pressure
MEWS	Modified Early Warning Score
mRNA	Messenger RNA
NRI	Net Reclassification Index
NRS	Normal Renin Sepsis
NYHA	New York Heart Association
OPN	Osteopontin
OR	Odds Ratio
PCZ	Procizumab (anti-DPP3 monoclonal antibody)
P/Fratio	PaO_2_/FiO_2_ ratio
PBS	Phosphate-Buffered Saline
PK/PD	Pharmacokinetics/Pharmacodynamics
RAAS	Renin–Angiotensin–Aldosterone System
RRT	Renal Replacement Therapy
ROS	Reactive Oxygen Species
SAPS II	Simplified Acute Physiology Score II
SI	Shock Index
SOFA	Sequential Organ Failure Assessment
SV	Stroke Volume
STEMI	ST-Elevation Myocardial Infarction
UI	Units of Intensity (oxidative stress measurement)
VO_2_	Oxygen Consumption

## Appendix A

Studies in Dpp3^−^/^−^ mice have revealed that complete loss of DPP3 causes a distinctive phenotype involving both metabolic and oxidative abnormalities [31]. Knockout animals exhibit elevated systemic oxidative stress, characterized by increased reactive oxygen species (ROS) levels, depletion of antioxidant enzymes, and impaired Nrf2/HO-1 signaling, confirming DPP3′s physiological role in redox regulation. These mice also show skeletal abnormalities and bone loss, associated with enhanced osteoclast differentiation and reduced osteoblast activity, indicating that DPP3 contributes to bone remodeling and mineral homeostasis [56]. Although cardiac morphology is generally preserved under baseline conditions, Dpp3^−^/^−^ mice display greater susceptibility to hemodynamic stress, with a blunted contractile response and faster decompensation under angiotensin II–induced pressure overload. Together, these findings suggest that DPP3 is not essential for basal viability but plays a protective role in oxidative balance and stress adaptation, whose absence predisposes to structural fragility and functional decline during cardiovascular or metabolic stress [31].
